# Presenting a multi-marker response-adapted framework for venetoclax duration in acute myeloid leukemia

**DOI:** 10.1007/s00277-026-07280-x

**Published:** 2026-09-14

**Authors:** Nabiha Adnan

**Affiliations:** Islamic International Medical College (MBBS), Islamabad, 46220 Pakistan

Dear Editor,

Acute Myeloid Leukemia (AML) is a progressively increasing form of leukemia with a consistently high level of incidence and mortality, now causing a global health strain. To such an extent that, by 2040, the number of cases is predicted to increase significantly to 184,287.88 and 165,537.59, respectively [1]. Resulting in an economic burden, a US analysis done by Hagiwara et al. on 9,455 newly diagnosed patients found that the average cost of healthcare per patient was $ 386,077 [2]. This emphasizes the urgent need for a system that can precisely evaluate treatment effectiveness and maximize its duration.

A recent retrospective, two-stage real-world cohort study conducted by Chen et al. evaluated early bone-marrow-guided venetoclax (VEN) management in patients with newly diagnosed AML who were unsuitable for intensive chemotherapy. Early marrow-guided treatment was associated with significantly reduced early VEN exposure and day-56 infection-related hospitalization (24.0% vs. 37.4%; adjusted OR 0.46, *p* = 0.005) in a sample of 371 evaluable patients. From the 237 responding patients, shortening VEN to ≤ 14 days per cycle decreased prolonged grade-4 neutropenia (41.6% vs. 61.0%; adjusted OR 0.41, *p* = 0.003) and infection-related hospitalization (adjusted OR 0.43, *p* = 0.014) without significant alteration in Measurable Residual Disease (MRD) negativity, overall survival, or relapse-free survival. These results imply that response-adapted VEN shortening might lessen toxicity without sacrificing effectiveness, but prospective validation is necessary due to the retrospective study and possible selection bias [3]. This study shows that early marrow morphology is functional but may be insufficient on its own; we propose combining it with MRD and molecular markers to guide VEN duration, so we can further optimize the balance between treatment efficacy and hematologic toxicity.

A recent study by Heusen et al. proposes MRD as a significant biomarker for prognostic, predictive, monitoring, and efficacy-response evaluations in acute myeloid leukemia (AML). It is also noted that a positive or negative MRD test result indicates whether or not quantifiable disease is found above particular thresholds, which can differ depending on the assay and laboratory and can be assessed as a complementary technique [4]. Further studies done by Bancos et al. discuss limitations of MRD as a single technique and propose a multimodal technique because each molecular marker, such as one marrow morphology, MRD, and molecular MRD via Next-Generation Sequencing(NGS), may be effective in diagnosing AML but has its own limitations and may not be effective in treating AML patients [5]. Thus, we propose combining early marrow morphology, MRD, molecular characteristics, and hematologic recovery into a *proposed multi-marker response-adapted framework* that could potentially provide a more precise basis for individualizing venetoclax duration. Furthermore, another review done by Samarkhazan et al. demonstrates the principle of multi marker/multi-omics assessment [6] that may help make a more accurate response-adapted approach possible by the integration of complementary molecular and transcriptomic indicators, enabling the customization of venetoclax duration based on both morphological and biological disease response, but it may have its own constraints. Therefore, we urge further research into the concept of a potential multi-marker panel that includes marrow morphology, MRD, and molecular MRD via NGS relevant molecular responses to identify a novel route to successful diagnosis. We also believe that certain aspects of Chen et al.‘s study have their own limitations. Firstly, the cohort study conducted is *not a randomized approach* but rather a retrospective one; this highlights the n*eed for more studies on this topic that use randomized groups*. Moreover, the author’s findings include the use of a single approach to reduce treatment, but AML is a complicated disease and may not be detected completely by a single marker. Lastly, the study shows that Chen et al. did not find any significant difference in *Overall Survival (OS)* or *Progression-Free Survival (PFS)* directing that the study was effective, but a more prospective study is needed to ensure that short VEN is truly applicable for effective treatment.

## Data Availability

No datasets were generated or analysed during the current study.
